# Peritoneal adhesion prevention with a biodegradable and injectable N,O-carboxymethyl chitosan-aldehyde hyaluronic acid hydrogel in a rat repeated-injury model

**DOI:** 10.1038/srep37600

**Published:** 2016-11-21

**Authors:** Linjiang Song, Ling Li, Tao He, Ning Wang, Suleixin Yang, Xi Yang, Yan Zeng, Wenli Zhang, Li Yang, Qinjie Wu, Changyang Gong

**Affiliations:** 1 State Key Laboratory of Biotherapy and Cancer Center, West China Hospital, Sichuan University, and Collaborative Innovation Center for Biotherapy, Chengdu, 610041, P. R. China

## Abstract

Postoperative peritoneal adhesion is one of the serious issues because it induces severe clinical disorders. In this study, we prepared biodegradable and injectable hydrogel composed of N,O-carboxymethyl chitosan (NOCC) and aldehyde hyaluronic acid (AHA), and assessed its anti-adhesion effect in a rigorous and severe recurrent adhesion model which is closer to clinical conditions. The flexible hydrogel, which gelated in 66 seconds at 37 °C, was cross-linked by the schiff base derived from the amino groups of NOCC and aldehyde groups in AHA. *In vitro* cytotoxicity test showed the hydrogel was non-toxic. *In vitro* and *in vivo* degradation examinations demonstrated the biodegradable and biocompatibility properties of the hydrogel. The hydrogel discs could prevent the invasion of fibroblasts, whereas fibroblasts encapsulated in the porous 3-dimensional hydrogels could grow and proliferate well. Furthermore, the hydrogel was applied to evaluate the anti-adhesion efficacy in a more rigorous recurrent adhesion model. Compared with normal saline group and commercial hyaluronic acid (HA) hydrogel, the NOCC-AHA hydrogel exhibited significant reduction of peritoneal adhesion. Compared to control group, the blood and abdominal lavage level of tPA was increased in NOCC-AHA hydrogel group. These findings suggested that NOCC-AHA hydrogel had a great potential to serve as an anti-adhesion candidate.

Post-operative peritoneal adhesions are regarded as inevitable and serious complications after surgeries such as cholecystectomy, gastrectomy, appendectomy, hysterectomy, colostomy, abdominoperineal resection, and abdominal vascular procedures^1^,^2^. Incidence of postsurgical adhesion usually ranges from 67% to 93% after general surgical abdominal procedures, and it is even up to 97% after open gynecologic pelvic operations^3^,^4^. Postsurgical adhesion not only brings huge financial burden to the patient’s families, but also results in many troubles, such as chronic pain, female infertility, bowel obstruction, and etc, to the patients^5^,^6^.

The peritoneum consists of only one layer of mesothelial cells and covers on the abdominal and pelvic cavities and the organs within^7^. Surgeries lead to tissue injury and ischemia, inducing a serious inflammatory response in which a fibrinous exudate is exuded into the injured peritoneum. Either the fibrinous exudates are completely degraded, resulting in normal peritoneal healing, or it persists and may serve as a scaffold for fibroblasts and endothelial cells in growth to form permanent fibrous peritoneal adhesions^8^,^9^. It is currently deemed that the intro-peritoneal fibrinolytic activity is dominated by the balance of tissue-type plasminogen activator (tPA) and plasminogen activator inhibitor 1 (PAI-1), which usually secreted by peritoneal mesothelial cell^10^. tPA leads to the conversion of plasminogen into plasmin that is highly effective in the degradation of fibrin into fibrin degradation products, however, PAI-1 antagonise and form inactive complexes with tPA^11^,^12^,^13^.

For decades, numerous efforts on post-surgery adhesion are focused on seeking effective approaches. One of the processes to avoid adhesions is the application of drugs such as anti-inflammatory and antithrombotic drugs. To be disappointed, due to its fast clearance in abdominal cavity and some side-effects, few of them can be applied to clinical practice^14^,^15^. Another approach is to apply mechanical barriers to separate the injured tissues from the injured areas during the healing process, and the barriers usually divided into two categories, liquid barriers (crystalloids, dextran, hyaluronic acid andicodextrin) and solid barriers (Interceed^®^, Preclude Peritoneal Membrane^®^, Seprafilm^®^ and SprayGel^®^)^16^,^17^,^18^. The liquid barriers’ persistent time is too short to keep their efficacy^18^. When it refers to solid barrier sheets, complete coverage of the injured areas cannot be easy, and it is difficult to handle and fix these materials to the damaged tissues, even some of them can aggressively adhere to any moisture such as the surgeon’s gloves during placement^19^,^20^,^21^. This may be a limitation for its further applications. It is therefore still a challenge to find a kind of anti-adhesion drug which is not only safe, effective, and easy to use, but also has an appropriate gelation and retention time.

In our previous works, we prepared a cross-linking hydrogel, which was based on biocompatible chitosan and hyaluronic acid^22^. The hydrogel was effective in reducing the abdominal adhesion in a sidewall defect-cecum abrasion model in rat. However, in clinic, once the abdominal adhesion formed, the patients must be received treatment again and again, because separating the adhesive tissue is so difficult that the corresponding adhesion preventions may not be satisfactory^23^. Therefore, we want to apply our biodegradable and injectable cross-linking hydrogel to a stricter recurrent adhesion rat model, in which the adhesion is not only stricter than side wall defect-cecum abrasion adhesion model but also closer to clinic. However, the results of pre-experiment showed an unsatisfactory anti-adhesion effect. So, in this study, we prepared an optimized NOCC-AHA hydrogel, which is more stable and stronger, for adhesion prevention in a rat repeated-injury model. Here, we characterized its morphology, degradation *in vitro* and *in vivo*, *in vitro* cell availability and effectiveness in preventing adhesions *in vivo*. Moreover, we also investigated the anti-adhesion mechanisms.

## Results and Discussion

### Characterization of AHA and NOCC

**Figure 1:**
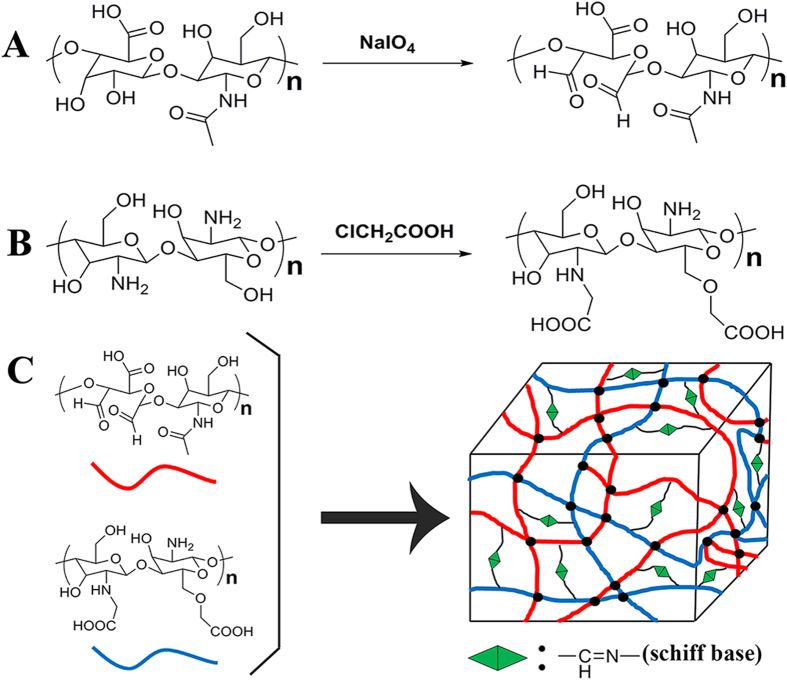
Synthesis of NOCC, AHA, and NOCC-AHA hydrogel. (**A**) synthesis of AHA; (**B**) synthesis of NOCC; (**C**) schematic illustration of the preparation of NOCC-AHA hydrogel by cross-linking NOCC with A-HA.

AHA was obtained by reacting HA with sodium periodate. NaIO_4_ oxidized the vicinal diol of the HA repeated unit to dialdehydes, and opening the sugar ring to get the dialdehyde derivative, AHA. The chemical structure of AHA was displaced in Fig. 1A, and the results of actual oxidation degree are listed in Table 1. Compared with our previous AHA, this actual oxidation degree was a little higher, so that there existed more aldehyde groups to participate the Schiff base reaction. NOCC was prepared by the introduction of carboxymethyl groups into N-terminal and O-terminal of chitosan to improve the water-soluble property of chitosan. The determined substitution degree of NOCC was 85%. The NOCC’s chemical structure was displaced in Fig. 1B.

NOCC and AHA were characterized by FTIR and ^1^H-NMR (Figure S1A and B). Compared with chitosan, NOCC showed an obvious and large absorption peak at 3434 cm^−1^, which corresponds to the carboxylic group of NOCC. By comparing with the chitosan, new signals at 2.7 ppm was observed in the spectrum of NOCC, which was reported in a previous study^24^. We could not find the difference between the FTIR spectra of HA and AHA. It is hypothesized that the formation of hemiacetals makes it difficult to detect the signal of the aldehyde group in the chain of AHA. Moreover, the ^1^H-NMR spectrum of AHA displayed two new peaks at 4.9 ppm and 5.0 ppm, which may correspond to hemiacetalic protons derived from the aldehyde groups and neighboring hydroxyl groups. The Schiff base reaction between amino of NOCC and aldehyde of AHA was accounted for the cross-linking between NOCC and AHA, which result in a cross-linked and porous NOCC-AHA hydrogel (Figure S1C).

### Rheological property

The rheological property of the NOCC-AHA hydrogels was measured at 37 °C by rheometer. To find a suitable anti-adhesion NOCC-AHA hydrogel, thirty-six hydrogel samples were designed by the orthogonal test. The results of gelation time and G’ were displayed in Table S1. As the Table S1 saying, with the theoretical oxidation degrees of AHA increasing, the gelation time was decreasing and G’ was bigger. Meanwhile, the concentration of NOCC and AHA showed a similar variation. As is well-known, too fast gelation rate will make it difficult to cover the damaged area completely before gelation; however, if the gelation time is too long and G’ is small, the flowing hydrogel cannot be adhered to the injured tissue tightly^25^. When theoretical oxidation degree of AHA was 40% (actual oxidation degree was 33.42% ± 0.58%), and NOCC (30 mg/mL) and AHA (30 mg/mL) were mixed with 1:1 ratio, the gelation time was 66 s and G’ is suitable for a more rigorous model such as repeated-injury adhesion model. Therefore, we applied this kind of hydrogel, which can easily cover the defects and adhere to the defects tightly, to do the next studies.

**Figure 2:**
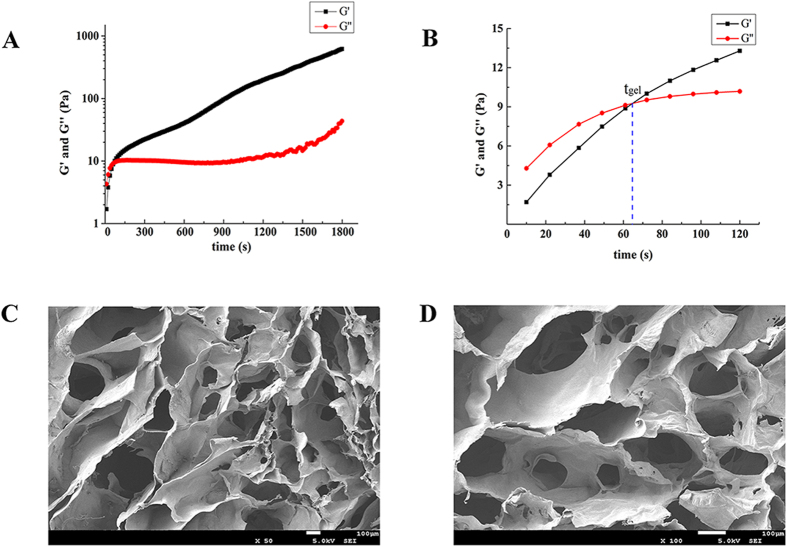
Rheological analysis and SEM evaluation of prepared NOCC-AHA hydrogel. (**A** and **B**) G’ and G” of NOCC-AHA hydrogel cross-linked by NOCC (30 mg/mL) and AHA (30 mg/mL). t_gel_ represents the crossover time point of G’ and G” curves; (**C** and **D**) SEM images of the hydrogel prepared by NOCC (30 mg/mL) and AHA (30 mg/mL) at 50× and 100×, respectively.

As displayed in Fig. 2A and B, the change in G’ and G” of NOCC-AHA hydrogel was a function of time. At the beginning, both G’ and G” were low, and G’ was lower than G”, which representing the sol state of the system. As time went on, Schiff bases between NOCC and AHA were formed, both G’ and G” were increased and G’ increased faster than G”. The gelation point (t_gel_) appears when G’ > G”. As the cross-linked gelation processing, the value of G’, which presents the intensity of hydrogel, was not only significantly larger than G”, but also larger than our previous NOCC-AHA cross-linked hydrogel. At the gelation point (t_gel_), G’ of NOCC-AHA was 9 Pa which was a little higher than our previous hydrogel (G’ was about 6 Pa at t_gel_). However, as the cross-linked gelation processing, the G’ of optimized hydrogel was much higher than our previous hydrogel. For example, G’ of NOCC-AHA was 200 Pa at 1200 seconds, which is much higher than our previous hydrogel (G’_t=1200s_ was about 90 Pa). So we applied the optimized NOCC-AHA hydrogel, which is more stable and stronger than our previous hydrogel, to adhesion prevention in a rat repeated-injury model.

### Morphology

The SEM micrographs of the cross-section of hydrogel, which prepared from NOCC (30 mg/mL) and AHA (30 mg/mL), were showed in Fig. 2C and D. As we can see, the hydrogel displayed a highly porous and interconnected interior structure, which may possess a high permeability for nutrients and support cellular growth. According to our previous studies^22^, the pore size was directly influenced by the cross-linking density, which related to the density of amino groups and aldehyde groups of the hydrogel.

### *In vitro* and *in vivo* degradation behavior

**Figure 3:**
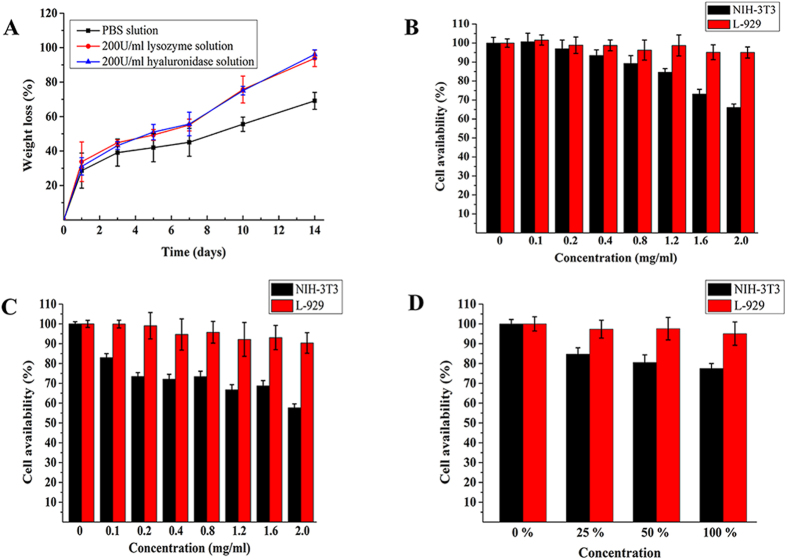
*In vitro* degradation and cell viability of NOCC-AHA hydrogel. (**A**) *In vitro* degradation kinetics of the hydrogel in PBS, 200 U/mL of lysozyme solution, or 200 U/mL of hyaluronidase solution at 37 °C. Data were presented as mean ± SD (n = 3); (**B,C** and **D**), cytotoxicity on NIH-3T3 cells and L-929 cells after 2 days incubation with NOCC, AHA and hydrogel extracts, respectively. Data were presented as mean ± SD (n = 6).

It is known that chitosan and hyaluronic acid is respectively depolymerized by lysozyme and hyaluronidase, which is widely distributed in human tissues. Therefore, we evaluated the *in vitro* degradation behavior of hydrogel, which was cross-linked by NOCC (30 mg/mL) and AHA (30 mg/mL), in simulated physiological conditions (PBS (pH = 7.4), hyaluronidase solution or lysozyme solution) at 37 °C. During the degradation process, we can see a significant decrease in sample volume, which may result from a fully contraction between degradation media and sample surface. As displayed in Fig. 3A, which was obviously observed is that the different degradation kinetics of hydrogels in hyaluronidase solution, lysozyme solution and PBS. The degradation property of first day was similar because of a requirement of swell for the freeze-dried hydrogel. Then, the hydrogel was degraded relatively rapidly in lysozyme solution (200 U/mL) and hyaluronidase solution (200 U/mL). Moreover, the degradation process of lysozyme solution and hyaluronidase were parallel and the hydrogels were degraded about 50% on 5^th^ day, and nearly completely degraded on 14 days. Meanwhile, the hydrogels were degraded about 70% by 14^th^ day in PBS. Eventhough, there existed no enzyme in PBS, the hydrogel discs might be degraded by hydrolysis. The degradation of hydrogel resulted from a combination of enzymolysis and hydrolysis. So we can draw a conclusion that the NOCC-AHA hydrogel has a good biodegradability. To be our interesting, we found that the degradation kinetics of NOCC-AHA hydrogel is lower than our previous hydrogel, it is because the optimized NOCC-AHA hydrogel was composed of higher oxidation degree of AHA and higher concentration of AHA and NOCC so that the optimized hydrogel was more stable and stronger.

**Figure 4:**
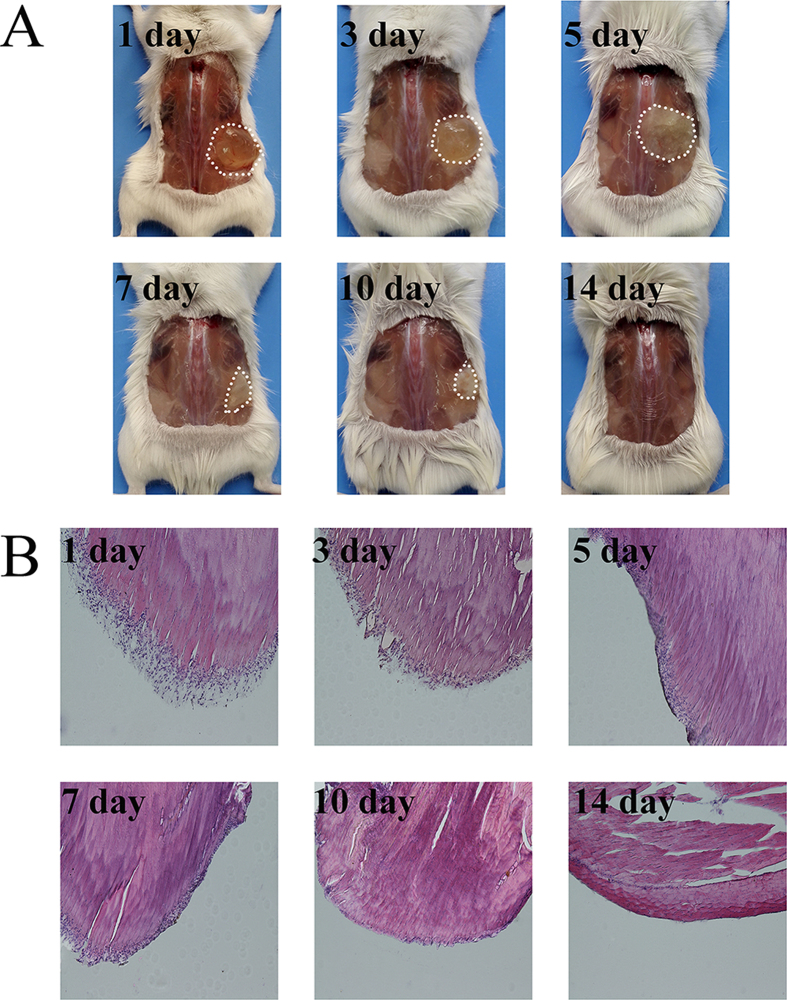
*In vivo* degradation behavior and biocompatibility of NOCC-AHA hydrogel. (**A**) *In vivo* observation of NOCC-AHA hydrogel degradation for different periods; (**B**) histological observations of biocompatibility assay at different time points.

As we all know, *in vivo* degradation time is a key index for anti-adhesion agent. *In vivo* degradation behavior of NOCC-AHA hydrogel was assessed by dorsal subcutaneous injection in BALB/c mice. As displayed in Fig. 4A, the transparent hydrogel was formed *in situ* after subcutaneous injection and the volume of hydrogel was decreased as time went on. The hydrogel degraded a little at the first 3 days, because the NOCC-AHA hydrogels consist of high concentration of NOCC and AHA and had a strong intensity. The hydrogel was degraded significantly at the 5^th^ day and spread around the injection site. This was not in accord with the results of *in vitro* degradation. It might be explained that there were many lipase, protease and enzyme in the body which different from *in vitro* condition. The hydrogel was disappeared at 14^th^ day. The quantification of the *in vivo* degradation of hydrogel was displayed in Figure S2. In addition, the histopathologic examination was employed to observe microscopic changes of the tissue around the injection site. As displayed in Fig. 4B, though there existed a lot neutrophils and macrophages in the tissue around the injection at the first 3 days, it decreased gradually and disappeared at 14^th^ day. Moreover, no hemorrhage, necrosis, or inflammatory exudate was observed. The *in vivo* degradation results showed that NOCC-AHA hydrogel maybe a potential safe anti-adhesion agent.

### *In vitro* cytotoxicity assessment

*In vitro* cytotoxicity of NOCC, AHA and hydrogel extract were assessed by cell viability on NIH-3T3 cells and L-929 cells using MTT method. As shown in Fig. 3B and C, when the input concentration of NOCC and AHA was 2 mg/mL, the viability of L-929 was higher than 90%, and the NIH-3T3 was higher than 60%. These results draw a conclusion that NOCC and AHA which have little toxicity were safe materials. As presented in Fig. 3D, when cultured in hydrogel extract, the viability of NIH-3T3 was higher than 80% and L-929 was approximately 100%. Figure S3 showed the cytotoxicity of the hydrogel at the 5th day. The hydrogel displayed none cytotoxicity at the 5th day. *In vitro* cytotoxicity results indicated that NOCC, AHA and the hydrogel extract had little toxicity, and the NOCC-AHA hydrogel could serve as a safe anti-adhesion material.

### Fibroblasts invasion test

**Figure 5:**
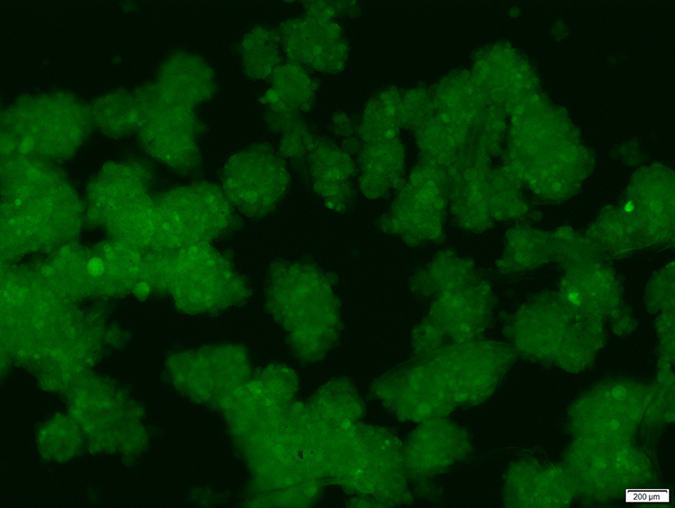
Fibroblasts on the surface of NOCC-AHA hydrogel after 24 h culture at 200×.

The adhesion of fibroblasts on the surface of the hydrogel discs was used to evaluate the prevention of fibroblasts invasion by NOCC-AHA hydrogel. What we can be seen from Fig. 5 was that the cells on the surface of hydrogel lost the polygonal morphology, floated and formed cell clusters. It suggested that fibroblasts couldn’t attach and grow on the surface of hydrogel. We wondered if the hydrogel’s toxic resulted in this phenomena. So, we employed the *in vitro* cytocompatibility assessment to measure the toxic of the hydrogel. Figure S4 showed that the fibroblast cells could grow well in the interior of hydrogel. Therefore, we concluded that the cytocompatible NOCC-AHA hydrogel can be developed for anti-adhesion.

### Anti-adhesion efficacy of NOCC-AHA hydrogel in rat repeated-injury adhesion model

In our previous works, the relatively low concentration and low actual oxidation of NOCC-AHA hydrogel was applied to a rat sidewall defect-cecum abrasion model, and received a satisfied anti-adhesion result. But, the previous hydrogel was not effective in the stricter repeated-injury adhesion model. In this study, we applied the optimized NOCC-AHA hydrogel, which was composed of high concentration of NOCC and AHA and possessed high intensity, to a stricter repeated-injury adhesion model, which usually occurred in clinic.

**Figure 6:**
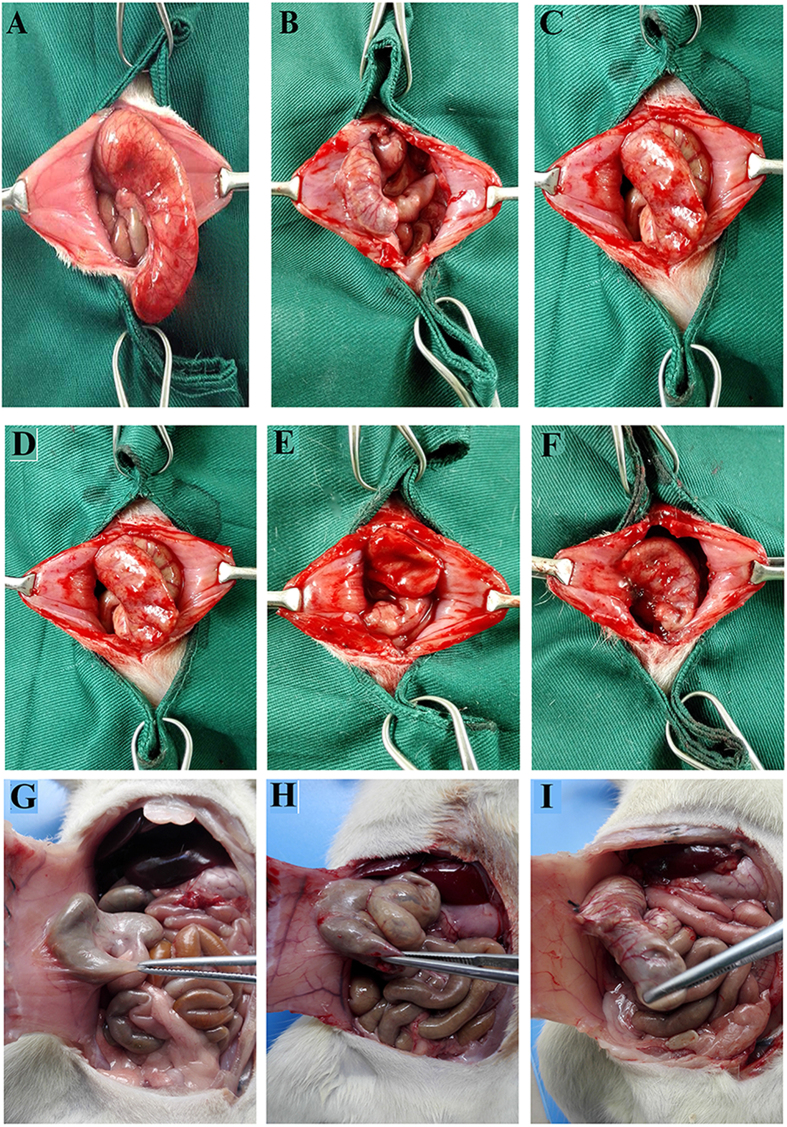
Establishment, treatment and anti-adhesion evaluation of rat repeated-injury adhesion model. (**A** and **B**) establishment of abdominal wall defect-cecum abrasion model; (**C**) a second laparotomy was performed to establish the repeated-injury adhesion model; (**D,E** and **F**) NS, HA hydrogel and NOCC-AHA hydrogel applied on the injured sites respectively; (**G**) score 5 adhesion occurred in NS-treated group on 14^th^ day after last surgery; (**H**) score 5 adhesion happened in the HA-treated group on 14^th^ day after last surgery; (**I**) no adhesions was found in NOCC-AHA hydrogel treated group two weeks after last surgery.

First of all, sidewall defect-cecum abrasion model was established as shown by Fig. 6A,B and C. Seven days later, a second surgery was conducted to separate adhesions by an appropriate dissection, and then the separated abdominal wall and cecal surface were re-abrade with a sterile gauze. Before suturing the enterocoelia, 1 mL of NS, HA hydrogel or NOCC-AHA hydrogel was applied to cover both the injured and the un-injured areas around (Fig. 6D,E and F). The abdominal cavity temperature accelerated the formation of NOCC-AHA hydrogel. The stable hydrogel was formed *in situ*. Then, the rats were sacrificed two weeks later to evaluate the anti-adhesion efficacy (Fig. 6G,H,I and Table 2). In NS group, all rats presented score 5 adhesion, which demonstrated the successful establishment of the repeated-injury adhesion model (Fig. 6G). HA hydrogel group had a mean score 5 adhesion, which displayed a poor anti-adhesion efficacy and had no significant difference compared with control group. Moreover, significantly lower adhesion score (P < 0.01) was found in NOCC-AHA hydrogel group. 5 of 7 rats were found no adhesion (Fig. 6I) and the other two rats developed score 1 adhesion. In addition, not only the defects were almost completely recovered within two weeks but also no NOCC-AHA hydrogel residue was found in abdominal cavity which demonstrated the high anti-adhesion efficacy and biodegradation of the NOCC-AHA hydrogel.

### Histopathological examination and SEM analysis

**Figure 7:**
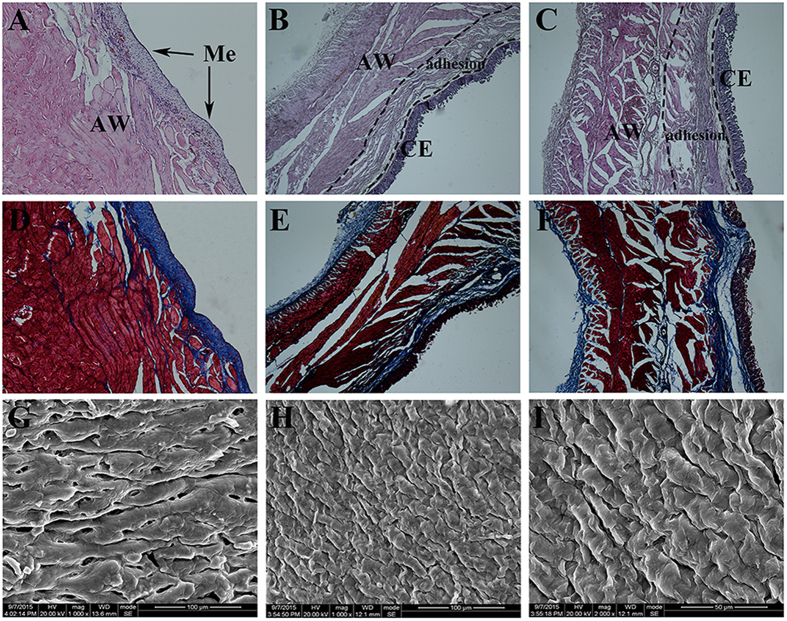
Histological evaluation and SEM images of tissues collected from rats treated with NS, HA hydrogel and NOCC-AHA hydrogel. (**A** and **D**) H&E and Masson staining of healed abdominal wall in the NOCC-AHA hydrogel group; (**B** and **E**) H&E and Masson staining of adhesion between cecum and abdominal wall from rat treated with HA hydrogel. (**C** and **F**) H&E and Masson staining of adhesion between cecum and abdominal wall from rat treated with NS. (**G**) SEM images (1000×) of surface of healed cecum treated with NOCC-AHA hydrogel; (**H** and **I**) SEM images (1000× and 2000×) of surface of healed peritoneum in NOCC-AHA hydrogel group. Me: mesothelial layer; CE: cecal mucosa; AW: abdominal wall.

For histological examination, tissue samples from damaged cecum and abdominal wall were stained with hematoxylin-eosin (H&E) and Masson trichrome staining. Severe adhesion between damaged abdominal wall and cecum could be observed in NS group (Fig. 7C and F) and HA hydrogel group (Fig. 7B and E), which abdominal wall and cecum were connected by various fibrous tissues and collagen deposition. However, for the group of NOCC-AHA hydrogel, the injured abdominal wall was filled with an integral neo-mesothelial cell layer with various subjacent fibrosis (Fig. 7A and D).

Moreover, SEM was also used to observe the healing of visceral peritoneal and cecum injury and morphology of mesothelial cells. 14 days after the administration of NOCC-AHA hydrogel, a layer of flattened, elongated, squamous-like cells which presented on the surface of neo-peritoneum and cecum were observed in NOCC-AHA hydrogel group (Fig. 7G,H and I), which revealed that the peritoneal injuries were completely recovered without the concerns of adhesion formation in the future.

### The anti-adhesion process of NOCC-AHA hydrogel

**Figure 8:**
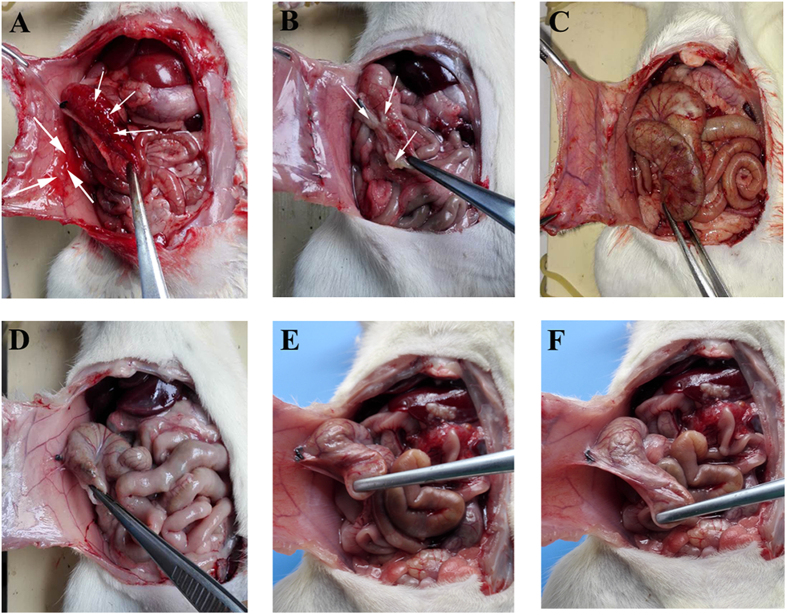
Observation of anti-adhesion process at 1 (**A**), 3 (**B**), 5 (**C**), 7 (**D**), 10 (**E**) and 14 (**F**) days after NOCC-AHA hydrogel treatment.

Rats treated with NOCC-AHA hydrogel were sacrificed on determined days. On the first day after NOCC-AHA hydrogel treated, the hydrogels which adhered on the injured surface of peritoneum and cecum could be observed clearly (Fig. 8A). The injured areas were inflamed obviously. Three days after treatment, the hydrogel was gradually degraded from the damaged area (Fig. 8B). On the fifth day, the hydrogel was almost disappeared (Fig. 8C). The inflamed surface of peritoneum and cecum were repaired on 5–7 days (Fig. 8D) and 7–10 days (Fig. 8E). On the 14^th^ day, completely recovered peritoneum and cecum could be seen with no adhesion (Fig. 8F). In conclusion, NOCC-AHA hydrogel was a effective anti-adhesion material in a rat repeated-injury model.

### Blood and abdominal lavage fluid level of tPA and PAI-1

The balance between fibrinolysis and antifibrinolytic activity, which was also expressed by the balance between tPA and PAI-1, has been demonstrated to be important in regulating the development of adhesions^26^,^27^. Moreover, an increase in tPA and/or a reduction in PAI-1 might result in an anti-adhesion effect. Though, It is generally acknowledged that the anti-adhesion efficacy of hydrogel was just owed to a physical barrier effect, which was independent of pharmacological mechanism, Yoon Yeo and etc 28 had been demonstrated that hyaluronic acid hydrogels and degradations could cause changes in mesothelial production of tPA and PAI-1. Therefore, the blood and abdominal lavage fluid levels of tPA and PAI-1 were measured by Enzyme Linked Immunosorbent Assay (ELISA) to clarify the relationship between levels of tPA and PAI-1 and anti-adhesion of NOCC-AHA hydrogel.

**Figure 9:**
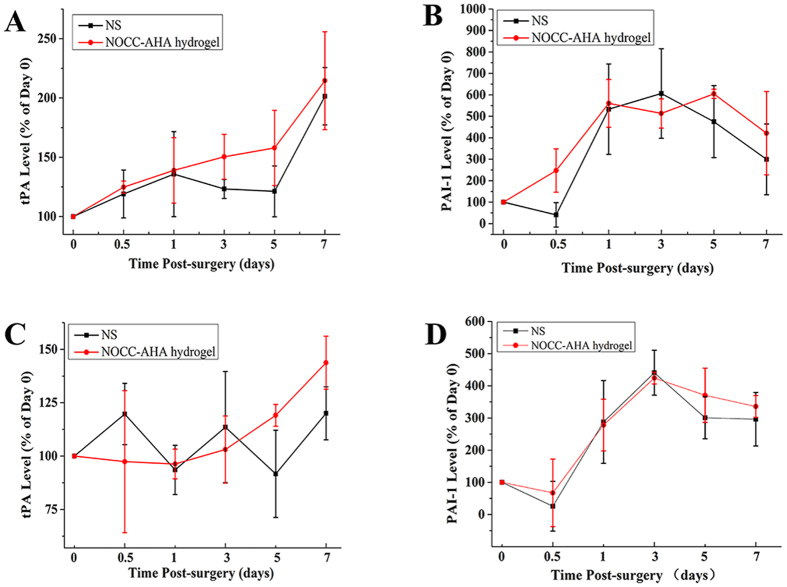
Blood and abdominal lavage fluid level of tPA and PAI-1. (**A** and **B**) the variation of tPA and PAI-1 level in abdominal lavage fluid after treated with NS and NOCC-AHA hydrogel. (**C** and **D**) the variation of tPA and PAI-1 level in blood after treated with NS and NOCC-AHA hydroge.

As shown in Fig. 9A and C, both tPA level in blood and abdominal lavage fluid were increased after treated with NOCC-AHA hydrogel with time going on. In NS group, the tPA levels in abdominal lavage fluid and blood were both slightly increased. What’s more, compared with NS group, the lavage fluid tPA level of NOCC-AHA hydrogel was higher in the determined time point, especially in day 3 and 5. The effect of increasing level of tPA in treated group might result from the wound healing and mesothelial proliferation promotion effect of hayaluronic acid^29^ and the hemostatic property^30^,^31^ and antimicrobial effect^32^ of chitosan, which resulted in a high anti-adhesion efficacy of NOCC-AHA hydrogel.

As expected, no differences of PAI-1 were found between NS group and NOCC-AHA hydrogel group in both blood and abdominal lavage fluid (Fig. 9B and D). However, there is a significant tendency that the level of PAI-1 increased at the first three days and decreased from the 5^th^ day. After a severe surgical injury, the level of PAI-1 increased at the initial time because some inflammatory reactions, then it fell into a normal level when both the adhesion had been formed and the injuries were healed^33^,^34^.

## Materials and Methods

### Materials and cell lines

Sodium hyaluronate (MW > 10^6^), chitosan (deacetylation degree (DD) of 85%), and 3-(4,5-dimethylthiazol-2-yl)-2,5-diphenyl-tetrazolium bromide (MTT) were purchased from SigmaAldrich (USA). NaIO_4_ was purchased from KeLong Chemicals (China). All other chemicals used were analytic grade without further purification.

NIH-3T3 cells and L-929 cells purchased from American Type Culture Collection (Rockville, Maryland) were separately cultured in RPMI-1640 medium and DMEM medium (Gibco, USA) with 10% fetal bovine serum (FBS) and antibiotics at 37 °C with a humidified 5% CO_2_ atmosphere, respectively.

Male Spraguee Dawley (SD) rats (250 ± 20 g), male and female BALB/c mice (18 ± 2 g), and green fluorescent female C57 mice (18 ± 2 g) were provided by HFK Bio-Technology Company (Beijing, China). All the animals were sex-separately housed in specific pathogen-free (SPF) conditions and were given ad libitum access to food and water. All animals would be in quarantine for a week before treatment.

### Ethics Statement

All animal work were conducted under the approved guidelines of Sichuan University (Chengdu, China) and approved by the Animal Care and Treatment Committee of Sichuan University (Chengdu, China).

### Synthesis and characterization of NOCC and A-HA

NOCC, and A-HA were synthesized as supplementary methods displayed, and they were characterized with proton Nuclear magnetic resonance spectroscopy (^1^H-NMR) (Varian 400 spectrometer, Varian Inc, Palo Alto, CA) and Fourier transform infrared (FTIR) spectroscopy (200SXV Infrared Spectrophotometer, Nicolet Co, Boston, MA).

### Preparation of NOCC-AHA hydrogel

The NOCC-AHA hydrogels were prepared by cross-linking between NOCC and AHA. In detail, NOCC was dissolved in normal saline (NS) at a concentration of 20, 25, 30 or 35 mg/mL, meanwhile, AHA was dissolved in NS at a concentration of 25, 30, 35 mg/mL. The cross-linked hydrogels were prepared through mixing NOCC and AHA solution at a volume ratio of 1:1.

### Characterization of NOCC-AHA hydrogel

The morphology of the NOCC-AHA hydrogel was tested by scanning electron microscopy (SEM). NOCC-AHA hydrogel was obtained by cross-linking at 37 °C and then freeze-dried. After that, the hydrogel was cryo-fractured in liquid nitrogen and the cross-sectional surface was coated with a thin layer of gold (the thickness of the gold layer is about 5–10 nm) before the measurement. The cross-sectional morphologies were observed using a scanning electron microscope (JSM-5900LV, JEOL, Japan).

Rheological characterization of the NOCC-AHA hydrogels were performed with HAAKE MARS RS6000 rheometer (Thermo Scientific, Germany) using parallel plate (P20 TiL) at 37 °C in oscillatory mode (τ = 1.000 Pa, f = 1.000 Hz, Gap = 1.000 mm, Volume = 0.4 mL, Duration = 30.00 min). The gelation time was considered as the time when storage modulus (G’) became higher than loss modulus (G”).

### *In vitro* and *in vivo* degradation behavior

The *in vitro* degradation properties of the NOCC-AHA hydrogels was carried out by simulated physiological conditions. In detail, 1 mL of the NOCC (25 mg/mL)-AHA (30 mg/mL) hydrogel was freeze-dried and weighted (W_0_). Then, the hydrogel discs were separated into 3 groups. Group 1, group 2, group 3 were incubated in 10 mL PBS (pH 7.4), 10 mL 200 U/mL lysozyme in PBS(pH 7.4), 10 mL 200 U/mL hyaluronidase in PBS (pH 7.4) respectively. Weight loss (ΔW) was recorded as a function of incubation time at 37 °C. The degradation media were refreshed every day to keep a continuous enzyme activity. At determined time points (1, 3, 5, 7, 10 or 14 days), the hydrogel discs were taken out from the degradation medium, freeze-dried and weighted (W_t_). After that, the freeze-dried discs were rehydrated, continued to degrade until the next time point. The weight loss percentage (ΔW%) at each time interval was calculated by the following formula:


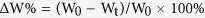


All results were obtained from the data of three individual experiments, and all data were expressed as the mean ± SD.

We employed BALB/c mice to evaluate the *in vivo* degradation behavior of the hydrogel. Briefly, 36 BALB/c mice were randomly separated into two groups: the treatment group and the control group. The treatment group was administered 400 μL NOCC-AHA hydrogel through dorsal subcutaneous injection and the control group was injected 400 μL NS correspondingly. Three mice of each group were killed at determined time (1d, 3d, 5d, 7d, 10, 14d). Meanwhile, the degraded conditions of the hydrogel were observed by opening the injection areas with a surgical scissor. Furthermore, the tissue around the injected site was collected, fixed in formalin for 72 h straightway. After that, the tissue was embedded in paraffin, sectioned, and stained with hematoxylin & eosin (H&E) for further histopathological examination.

### Cytotoxicity assessment

*In vitro* cytotoxicity of NOCC, AHA, and NOCC-AHA hydrogel extract were assessed by the MTT assay using NIH-3T3 cells and L-929 cells. The NIH-3T3 cells and L-929 cells were seeded into 96-well plates. After incubation 24 h, the medium was replaced with a series concentration (0, 0.1, 0.2, 0.4, 0.8, 1.2, 1.6, 2.0 mg/mL) of NOCC or AHA in incubation medium for another 48 h or 120 h, respectively. The cytotoxicity of NOCC-AHA hydrogel extracts was investigated as described below. Briefly, 1 mL of the NOCC-AHA hydrogel was extracted by 10 mL DMEM with 10% FBS for 24 h at 37 °C. Then, the extracted solution was sequential diluted to get different concentrations of the leachates. After that, NIH-3T3 cells and L-929 cells were incubated with the extracts of different concentrations (25%, 50%, 100%) for another 48 h respectively. Subsequently, 20 μL of MTT (5 mg/mL) was added to each well, and then incubated at 37 °C for another 4 h. Dissolving the precipitated formazan by 150 μL DMSO and measured the absorbance at 570 nm. All experiments were carried out in triplicate. Results were expressed as the mean ± SD of the measured absorbance which normalized to the absorbance of non-treated control cells in plain medium.

### Prevention of fibroblasts invasion

We applied primary murine fibroblasts which isolated from new-born green fluorescent C57 mice to assess the prevention of fibroblasts invasion of the NOCC-AHA hydrogel. Briefly, the fibroblasts were cultured in DMEM with 10% FBS, and fibroblasts at passage 3 were used in this experiment. To evaluate the prevention of fibroblasts invasion for NOCC-AHA hydrogel, 600 μL of hydrogel was added to the 24-well plate for thoroughly covering the bottom of the well. The hydrogels were incubated at 37 °C for 1 h to form a stable hydrogel disc (the thickness of the disc was about 3mm) which adhered to the bottom of wells, and washed with 1 mL DMEM medium. Subsequently, about 30000 fibroblast cells suspended in DMEM medium with 10% FBS were seeded into each well. After incubation for 24 h, the morphology of fibroblast cells was observed by Leica fluorescence microscope.

### Evaluation of anti-adhesion efficacy of NOCC-AHA hydrogel in the rat repeated-injury model

The adhesion prevention efficacy of NOCC-AHA hydrogel was assessed in a strict recurrent adhesion rat model^35^,^36^. Primarily, abdominal wall defect-cecum abrasion intraperitoneal adhesion model was developed. Briefly, rats were anesthetized with a single intraperitoneal injection of chloral hydrate (10%, 3 mL/kg). The abdomen was shaved and sterilized with iodine and alcohol solution. Then, the peritoneal cavity was opened with a 4 cm long, anterior midline incision and the cecum was identified and abraded with a 2 cm^2^ defect by rubbing with sterile dry medical gauze. Meanwhile, a 1 × 1 cm apposing parietal peritoneal damage with punctate hemorrhage was created by dry gauze. Then the two damaged areas was joined together with 3/0 silk suture for induce adhesions due to the floppy cecum in rats. Subsequently, the incision was closed in two layers (peritoneum layer and skin layer) with 4/0 medical silk suture. Seven days later, a second surgery was performed to cut the adhesions by a appropriate dissection. The separated cecal and abdominal wall surfaces were abraded again with sterile gauze until a hemorrhagic surface was appeared. Before suturing the enterocoelia, 1 mL of normal saline (NS), HA hydrogel (commercially used), NOCC-AHA hydrogel (7 rats per group) was applied to cover both injured areas and the un-injured surfaces around. For the group of NOCC-AHA hydrogel, 0.5 mL of NOCC (30 mg/mL) and 0.5 mL of AHA (30 mg/mL) were placed in separate sterile 2 mL syringes, which were equipped with a Baxter dual valve applicator, co-extruded through a 15-gauge needle and easily coated on the damaged cecum surface and the injured abdominal wall. The hydrogel on the defected surfaces was allowed to thoroughly congeal (about 2–3 min). In order to ensure the sterilization of NOCC-AHA hydrogel, NOCC and AHA stock solutions were sterilized by filtered it with sterile syringe filter (0.22 μm, Millipore).

Two weeks after the last surgery, the rats were sacrificed with an overdose of intravenous sodium pentobarbital. The efficacy of adhesion was assessed by the standard adhesion scoring system^37^, which has been widely used in this field. The scores of adhesion were taken by a double-blind process.The injured cecum, injured abdominal wall, and adhesion tissues comprising of abdominal wall and cecum were obtained and fixed in formalin for 72 h straightway. Then, the tissue was embedded in paraffin, sectioned, and stained with Hematoxylin & Eeosin and Masson trichrome for further histopathological examination. For SEM examination, the collected tissues were fixed with 2.5% glutaraldehyde in PBS immediately, gradient dehydrated with ethanol and examined using a scanning electron microscope (JSM-5900LV, JEOL, Japan).

### The anti-adhesion process of NOCC-AHA hydrogel

The anti-adhesion process of NOCC-AHA hydrogel was further explored. The above procedures were repeated. After treated with NOCC-AHA hydrogel, rats were dissected and dynamically observed at 1, 3, 5, 7, 10 and 14 days. At determined time, three rats were sacrificed and observed the effect of adhesion and the degradation of NOCC-AHA hydrogel as well.

### Effects of NOCC-AHA hydrogel on tPA and PAI-1 production in blood and abdominal lavage fluid

36 rats were divided into two groups: NOCC-AHA hydrogel group and NS group, and the above procedures were repeated. At predetermined time (0, 0.5, 1, 3, 5 and 7 days), three rats of each group were sacrificed with an overdose of inhalational ether after the blood samples (1 mL) were collected from orbit. The blood samples were immediately centrifuged at 5000 g for 20 min to separate plasma, which was then frozen at −80 °C until analysis. Then, 3 mL 0.9% NaCl solution was injected through a small access of 2 cm on the right side of the abdomen and the abdomen was gently shaken for 1 min to achieve fully irrigation^38^. Afterward, a sample of the abdominal lavage fluid(1 mL) was taken with a syringe, centrifuged at 2500 g for 20 min to separate impurities and frozen at −80 °C until use. tPA and PAI-1 levels in each sample were determined by rat tPA total ELISA kit and rat PAI-1 total ELISA kit (HaiTaiTongDa Tech Co., Ltd Beijing China).

### Statistical analysis

The statistical analysis was carried out using SPSS 15.0 software (Chicago, IL, USA). Adhesion scores did not always follow a normal distribution, therefore statistical inferences were made using Mann-Whitney U-tests, or Fisher’s exact test. A P value < 0.05 on a 2-tailed test was considered statistically significant.

## Conclusions

The biodegradable and injectable *in situ* cross-linking NOCC-AHA hydrogel described here are easy to use and highly effective in anti-adhesion in the severe repeated-injury rat model. The safe hydrogel is none cytotoxicity, biodegradation and biocompatibility. In a more rigorous recurrent adhesion rat model, the NOCC-AHA hydrogel showed a significant anti-adhesion efficacy, which makes it promising to serve as a potential anti-adhesion candidate.

## Additional Information

**How to cite this article**: Song, L. *et al.* Peritoneal adhesion prevention with a biodegradable and injectable N,O-carboxymethyl chitosan-aldehyde hyaluronic acid hydrogel in a rat repeated-injury model. *Sci. Rep.* **6**, 37600; doi: 10.1038/srep37600 (2016).

**Publisher’s note**: Springer Nature remains neutral with regard to jurisdictional claims in published maps and institutional affiliations.

## Supplementary Material

Supplementary Information

## Figures and Tables

**Table 1:** Oxidation degree of aldehyde hyaluronic acid (AHA).

Samples	Theoretical oxidation (%)	Actual oxidation degree (%)
AHA-1	30	22.05 ± 1.11
AHA-2	40	33.42 ± 0.58
AHA-3	60	36.42 ± 1.16

**Table 2:** Anti-adhesion of NOCC-AHA hydrogel in a rat repeated-injury adhesion model (**P < 0.01).

Adhesion	Control (n = 7)	HA hydrogel (n = 7)	NOCC-AHA hydrogel (n = 7)
Score 5	7	5	0
Score 4	0	2	0
Score 3	0	0	0
Score 2	0	0	0
Score 1	0	0	2
Score 0	0	0	5
Median score	5	5	0**
