# Supplementary material for: Peritoneal adhesion prevention with a biodegradable and injectable N,O-carboxymethyl chitosan-aldehyde hyaluronic acid hydrogel in a rat repeated-injury model
Source: Sci Rep. 2016 Nov 21;6:37600. doi: 10.1038/srep37600 (PMC5116612; doi:10.1038/srep37600)
Supplement: Supplementary Information [file srep37600-s1.doc]

**Peritoneal adhesion prevention with a biodegradable and injectable N,O-carboxymethyl chitosan-aldehyde hyaluronic acid hydrogel in a rat repeated-injury model**

Linjiang Song^+^, Ling Li^+^, Tao He, Ning Wang, Suleixin Yang, Xi Yang, Yan Zeng, Wenli Zhang, Li Yang^*^, Qinjie Wu^*^, Changyang Gong^*^

State Key Laboratory of Biotherapy and Cancer Center, West China Hospital, Sichuan University, and Collaborative Innovation Center for Biotherapy, Chengdu, 610041, P. R. China

^+^ These authors contributed equally to this work.

^*^ To whom should be corresponded (C Gong, Q Wu, and L Yang). E-mail: [chygong14@163.com](mailto:chygong14@163.com), [cellwqj@163.com](mailto:cellwqj@163.com), or [yangli@scu.edu.cn](mailto:yangli@scu.edu.cn).

**S1. Synthesis of NOCC and AHA**

NOCC was synthesized according to our previous works.[^1^](#_ENREF_1) In detail, 10 g of chitosan was dissolved in 75 mL of isopropanol and the slurry was stirred in a round bottom flask at room temperature. 25 mL 10 N NaOH was added dropwise to the stirred slurry in 30 minutes. After stirred 30 minutes later, 20 g monochloroacetic acid was added dropwise to the alkaline slurry above in 20 minutes. Then the solution was stirred at 60 ^o^C for 3 h. Subsequently, the reaction mixture was filtered to gain the crude products (NOCC). The pure NOCC was obtained by washed with 80% methanol, alcohol and vacuum-drying.

AHA was prepared by periodate oxidation method according to our previous works with a little improvement.[^1^](#_ENREF_1) Briefly, 1 g (2.5mmol repeated units) HA was completely dissolved in 100 mL distilled deionized (dd) water. Then, 160 mg (0.748 mmol), 210 mg (0.982 mmol) or 320 mg (1.496 mmol) NaIO_4_ in 5 mL dd water was added dropwise to the HA solution to achieve various oxidation degrees. Then, the mixture was stirred in the dark at room temperature for 2.5 h, followed by adding 500 μL ethylene glycol to quench the reaction. After stirring for another 1 h, the solution was dialysised (MWCO 10000) against distilled water for 3 days. The water was changed by every 8 h during the dialysis period. The dry product (AHA) was obtained by freeze drying. The actual oxidation degree of AHA was quantified by calculating the number of aldehyde groups in the polymer by trinitrobenzene sulfonate (TNBS) assay.[^1-3^](#_ENREF_1)

**S2. *In vitro* cytocompatibility**

Briefly, 200 μL of fibroblast/medium suspension was mixed with 200μL AHA stock solution. Furthermore, 400 μL of NOCC (30 mg/mL) was added. The resulting solutions (the cell counts were about 1 × 10^6^) were gently mixed and added to the 24-well plate. A composite cells/hydrogel matrix was formed after incubation at 37 ^o^C for 30 minutes. Subsequently, 1 mL of DMEM medium with 10% FBS was added in the surface of the matrix. After incubation at 37 ^o^C for 24 h, the morphology of fibroblast inside the hydrogel was viewed by Leica fluorescence microscope. All experiments were carried out in triplicate.

**Supporting Table S1**

**Table S1** rheological results of different types of NOCC-AHA hydrogel

| Theoretical oxidation degree | Concentration of A-HA (mg/ml) | Concentration of NOCC  (mg/ml) | gelation time (s) | G’ (pa)  t=600s | G’ (pa)  t=1800s |
| --- | --- | --- | --- | --- | --- |
| 30% | 25 | 20 | 240 | 5.148 | 47.04 |
|  |  | 25 | 147 | 9.340 | 60.02 |
|  |  | 30 | 66 | 21.73 | 223.0 |
|  |  | 35 | 24 | 123.8 | 1366 |
|  | 30 | 20 | 96 | 8.806 | 56.95 |
|  |  | 25 | 144 | 18.34 | 255.6 |
|  |  | 30 | 54 | 37.21 | 228.6 |
|  |  | 35 | 12 | 72.48 | 2113 |
|  | 35 | 20 | 420 | 5.75 | 132.2 |
|  |  | 25 | 102 | 22.83 | 129.2 |
|  |  | 30 | 96 | 23.30 | 136.8 |
|  |  | 35 | ----- | 61.49 | 158.6 |
| 40% | 25 | 20 | 138 | 8.9 | 48 |
|  |  | 25 | 108 | 11.86 | 62.1 |
|  |  | 30 | ----- | 16.56 | 53.40 |
|  |  | 35 | ----- | 17.41 | 109.6 |
|  | 30 | 20 | 252 | 11.68 | 92.08 |
|  |  | 25 | 102 | 21.74 | 224.7 |
|  |  | 30 | 66 | 40.21 | 618.7 |
|  |  | 35 | ----- | 102 | 1288 |
|  | 35 | 20 | 96 | 15.17 | 179.2 |
|  |  | 25 | 66 | 28.00 | 315.3 |
|  |  | 30 | 42 | 50.47 | 581.8 |
|  |  | 35 | ----- | 81.83 | 896 |
| 60% | 25 | 20 | 54 | 18.68 | 224.2 |
|  |  | 25 | 18 | 54.27 | 472.5 |
|  |  | 30 | ----- | 37.21 | 148 |
|  |  | 35 | ----- | 99.61 | 1289 |
|  | 30 | 20 | 24 | 19.51 | 214.9 |
|  |  | 25 | 12 | 52.17 | 2683 |
|  |  | 30 | ----- | 100.0 | 1443 |
|  |  | 35 | ----- | 119.3 | 1508 |
|  | 35 | 20 | ----- | 26.11 | 459.0 |
|  |  | 25 | ----- | 79.52 | 795.9 |
|  |  | 30 | ----- | 74.82 | 702.4 |
|  |  | 35 | ----- | 90 | 273 |


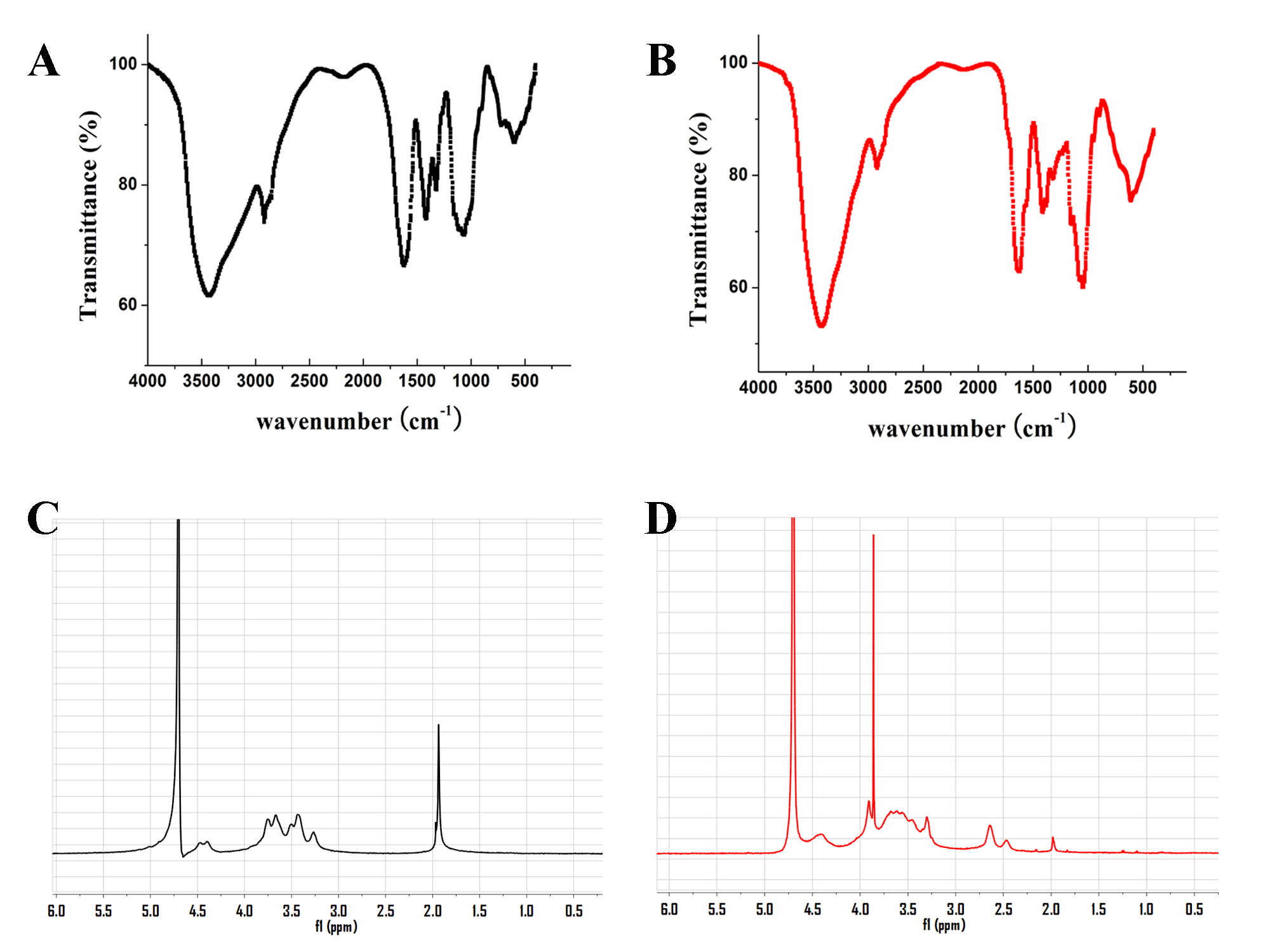


**Figure S1**. FTIR spectra of NOCC (A) and AHA (B). 1-NMR spectra of NOCC (C) and AHA (D).


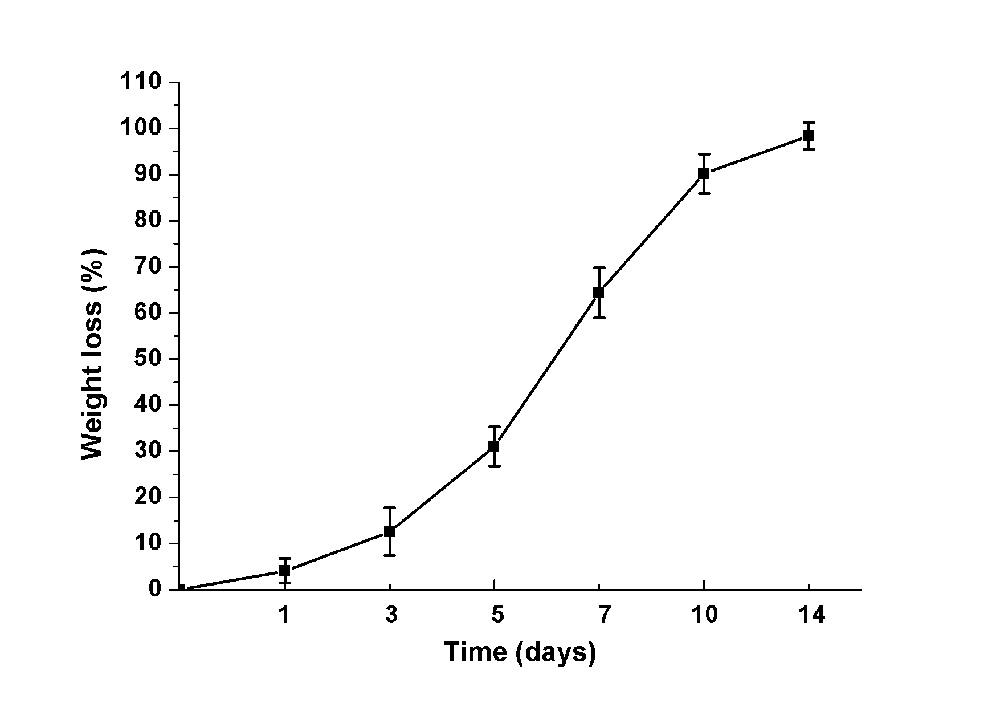


**Figure S2.** The quantification of the in vivo degradation of hydrogel

**
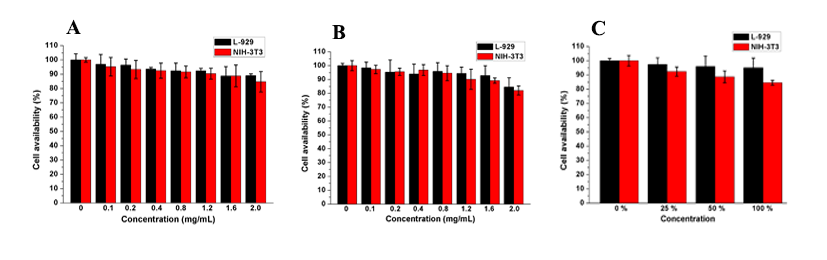
**

**Figure S3.** Cytotoxicity on NIH-3T3 cells and L-929 cells after 5 days incubation with NOCC (A), AHA (B) and hydrogel extracts (C), respectively. Data were presented as mean ± SD (n = 6).


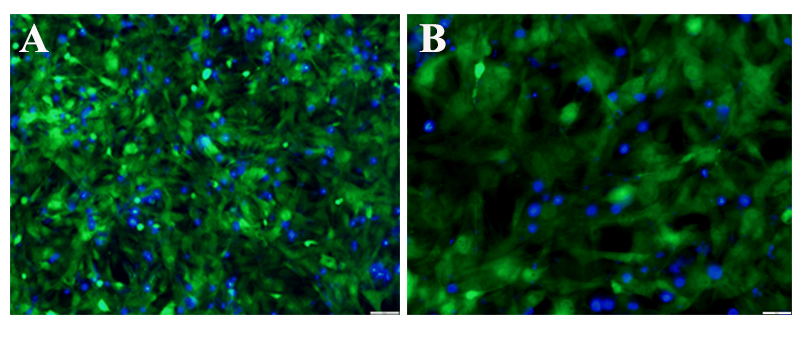


**Figure S4.** *In vitro* cytocompatibility of NOCC-AHA hydrogel. A and B, the morphology of the encapsulated fibroblasts in NOCC-AHA hydrogel after 24 h culture at 100× or 200×, respectively. Cell nuclei were dyed by DAPI (blue).

**References**

1. Li, L. *et al.* Biodegradable and injectable in situ cross-linking chitosan-hyaluronic acid based hydrogels for postoperative adhesion prevention. *Biomaterials* **35**, 3903-3917, (2014).

2. Su, W. Y., Chen, Y. C. & Lin, F. H. Injectable oxidized hyaluronic acid/adipic acid dihydrazide hydrogel for nucleus pulposus regeneration. *Acta biomater.* **6**, 3044-3055, (2010).

3. Prestwich, G. D., Marecak, D. M., Marecek, J. F., Vercruysse, K. P. & Ziebell, M. R. Controlled chemical modification of hyaluronic acid: synthesis, applications, and biodegradation of hydrazide derivatives. *J. Control. Release* **53**, 93-103, (1998).
